# An introduction to male breast cancer for urologists: epidemiology, diagnosis, principles of treatment, and special situations

**DOI:** 10.1590/S1677-5538.IBJU.2021.0828

**Published:** 2022-03-15

**Authors:** Fabiana Baroni Alves Makdissi, Silvana S. Santos, Almir Bitencourt, Fernando Augusto Batista Campos

**Affiliations:** 1 AC Camargo Cancer Center Centro de Referência da Mama São Paulo SP Brasil Centro de Referência da Mama, AC Camargo Cancer Center, São Paulo, SP, Brasil

**Keywords:** Breast Neoplasms, Male, Diagnostic Imaging, Genetics, Transgender Persons

## Abstract

Breast cancer (BC) is mainly considered a disease in women, but male BC (MaBC) accounts for approximately 1.0% of BC diagnoses and 0.5% of malignant neoplasms in the western population. The stigmatization of MaBC, the fact that men are less likely to undergo regular health screenings, and the limited knowledge of health professionals about MaBC contribute to men being diagnosed at more advanced stages. The aim of this article is to increase the visibility of MaBC among urologists, who have more contact with male patients. This review highlights key points about the disease, the risk factors associated with MaBC, and the options for treatment. Obesity and increased population longevity are among the important risk factors for MaBC, but published studies have identified family history as extremely relevant in these patients and associated with a high penetrance at any age. There is currently no screening for MaBC in the general population, but the possibility of screening in men at high risk for developing BC can be considered. The treatment of MaBC is multidisciplinary, and, because of its rarity, there are no robust clinical studies evaluating the role of systemic therapies in the management of both localized and metastatic disease. Therefore, in current clinical practice, treatment strategies for men with breast cancer are extrapolated from information arising from studies in female patients.

## INTRODUCTION

Breast cancer (BC), despite being commonly considered a disease in women, can also affect men. However, male BC (MaBC) is rare, accounting for approximately 1.0% of BC cases and 0.5% of malignant neoplasms in the general western population (1, 2). It was estimated that 2650 men in North America will be diagnosed with BC in 2021, and 530 men are expected to die from the disease (1). Data on the incidence of MaBC in other countries, especially in developing countries, are scarce.

In Brazil, the most common cancers in men, excluding non-melanoma skin cancer, are prostate and colorectal cancers. Despite its rarity, it is estimated that MaBC accounts for at least 1% of all BC cases in the country (3). As 66,680 women are expected to be diagnosed with BC in 2021, it is estimated that more than 600 men will be diagnosed with BC in Brazil. Although we do not have information on incidence trends in Brazil, it is known that the incidence of MaBC has increased worldwide. In 1975, there were 0.85 cases per 100,000 men in the general population, and in 2011, this number had increased to 1.43 (4).

Older studies have shown that the interval between symptom onset and the diagnosis of BC in men can reach 21 months, whereas more recent studies have shown that this duration can still be as long as 12 months (5). Historically, men have greater resistance to accessing medical services and health prevention programs, which leads to greater diagnostic delays and higher overall mortality than in women (6). In the case of BC, in addition to peculiar male behavior toward health (no diseases prevention or regular routine exams), the stigmatization of MaBC and the lack of knowledge by health professionals contribute to diagnosis at more advanced stages in men (5). In a Brazilian study conducted among university students and professors about their knowledge about cancer, when asked about the possibility of men developing BC, 30.6% believed that men could not develop BC. When asked which cancers were the most prevalent in men, only 3.0% of responses included breast as a possible site (7). In another study, 25.8% of men had no knowledge about MaBC. Only 8.1% had received any professional guidance about the disease, 61.0% did not know about the need for self-examination, and 39.0% did not know how to perform self-examination (8).

The objective of this article is to increase the visibility of MaBC among urologists, as they have more contact with male patients. Here, we highlight key points about MaBC, the associated risk factors, and the options for treatment. In addition to individual factors, an increasing number of studies have shown that genetic predisposition seems to play a very important role in these patients, and this knowledge is essential for professionals to improve care for patients and their families. By expanding the information to these professionals, we hope to improve access to information and promote quality primary care for the entire population (9).

### Epidemiology and Risk Factors

The risk factors for female BC (FBC) are widely known. The most well-known risk factors are those that increase the time and concentration of female hormones active in the breast tissue over time. These include age (with increased incidence from 40 years old and peak incidence over 50 years old), early menarche, late menopause, indiscriminate use of hormone replacement therapy, nulliparity, late age at first pregnancy (over 30 years old), and contraceptives (10). In addition, chest exposure to ionizing radiation and a family history of BC are important risk factors associated with FBC.

There are also known risk factors for MaBC, including obesity and increased population longevity (3, 10); however, the impact of risk factors is less known, as MaBC is less frequent than FBC and less studied.

Factors associated with an increased risk of MaBC that should be known by healthcare professionals who see these patients on a regular basis are: general (gynecomastia, increased blood estradiol, liver disease, obesity, testicular abnormalities, Klinefelter syndrome, ionizing radiation, longevity) and familiar/hereditary (family history for BC, hereditary cancer-related syndromes). However, some men without these risk factors still develop CM, although in some cases there may be uninformed, unknown or incorrectly collected data. There was an increase in the incidence of MaBC in the United States from 1973 to 1998; however, associated risk factors were not analyzed (11). In a study of 49 American patients at a single institution (12), 75.5% of patients did not have any of the general risk factors listed above, but 44.0% had a family history of MC.

The existing studies indicate that risk factors linked to family history are highly relevant in this group of patients, and this information has the potential to change the entire approach when a man is diagnosed with BC, including assessing risk with the patient’s family (11, 12). When a patient is diagnosed with cancer, treatment planning and follow-up are centered on the individual; however, the family approach generates changes in the care protocol when considering the possibility of identifying a genetic alteration that increases the risk of disease.

According to the National Comprehensive Cancer Network (NCCN) criteria (10) (2021), MaBC is a criterion for testing high-penetrance genes at any age, especially *BRCA1*/2, CDH1, PALB2, PTEN, and p53. According to this increased risk, there is an indication for self-examination and clinical examination of the breasts starting at age 35. According to the recommendations followed by the AC Camargo Cancer Center do Brasil (13) for MC testing, it is necessary to refer for genetic counseling:

- Breast cancer ≤45 years
- Breast cancer ≤50 years and one of the following:
  b 1) Second primary breast cancer
  b 2) Family with breast cancer at any age
  b 3) ≥1 family member with pancreatic cancer
  b 4) ≥1 family member with prostate cancer (Gleason score ≥ 7)
  b 5) Limited family history (<2 1st- or 2nd-degree female relatives alive up to 45 years old)
- Triple-negative breast cancer ≤60 years
- Breast cancer at any age if:
  d 1) ≥2 family members with breast, pancreatic, or prostate cancer (Gleason score ≥7)
  d 2) ≥1 family member with breast cancer <50 years
  d 3) ≥1 family member with ovarian cancer
  d 4) ≥1 family member with male breast cancer
  d 5) Ashkenazi ancestry
- Male breast cancer
- Patient without breast cancer, but based on family history (discuss interpretation limitations for the family):
  f 1) 1st- or 2nd-degree relative meeting the testing criteria

Epigenetic events also play an important role in CMM carcinogenesis, often associated with earlier age at diagnosis, and these changes are associated with worse prognosis (3).

*ATM, BRCA1, PALB2, RAD51B*, and *XRCC3* have epigenetic signatures in MaBC that are absent in corresponding normal tissue and gynecomastia samples obtained from patients without cancer or with a family history of BC. The *RAD51B* and *XRCC3* signatures can accurately discriminate MaBC from gynecomastia, with normal breast tissues showing methylation of these gene promoters at lower levels than in MaBC, suggesting the existence of a tumorigenesis pathway (3, 14).

Unlike *BRCA1* mutation, *BRCA2* alteration is more common in MaBC than in FBC. In high-risk families, about 60-70% of MaBC cases have a *BRCA2* alteration. The risk of BC in patients with such alterations is 5.0-10.0%, whereas the risk of MaBC in the general population is 0.1%. The MaBC phenotype in carriers of this mutation includes high-grade, progesterone receptor-negative, HER2-enriched tumors, and patients tend to be younger (< 50 years) (3, 15).

## DIAGNOSIS

**Figure 1:**
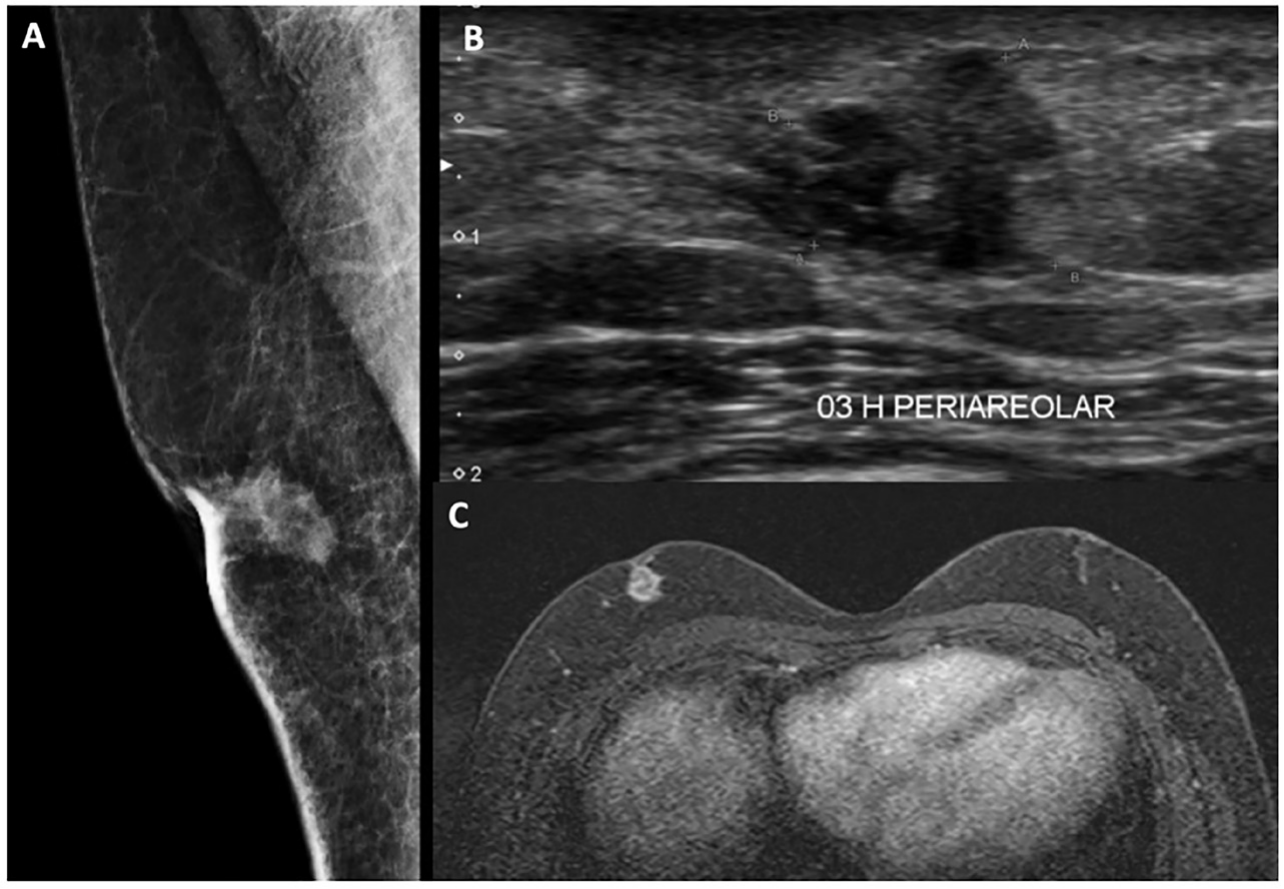
An irregular mass in the retroareolar region of the right breast in a 44-year-old man, seen on mammography (A), ultrasonography (B), and magnetic resonance imaging (C).

For women, the lifetime risk of BC is one in eight, whereas for men, the lifetime risk is one in a thousand (4, 5). Mammography (16) plays a key role in routine screening and reducing mortality in women, especially when performed annually; however, the low incidence of MaBC does not allow the indication of periodic examinations (see Figure-1). However, the possibility of screening in men at high risk for developing BC, such as those with mutations in the BRCA genes, is currently being discussed (17). Currently, the NCCN guidelines recommend that men with BRCA mutations receive training and education in breast self-examination and undergo annual clinical breast examination starting at 35 years of age. Therefore, annual screening mammography can be considered in men with gynecomastia starting at age 50 or in men with a family history of BC 10 years before the age at diagnosis of the youngest known case in the family (10).

In a study of 3,806 MaBC cases and 571,115 FBC cases, the mean age at diagnosis was higher in men than in women (64.2 years vs. 59.1 years, p<0.01) (18). Further, men were more likely to have lymph node metastasis than women (N1mic: 5.9% vs. 4.0%; N1-3: 12.1% vs. 8.7%; N0: 73.3% vs. 80.1%; all p<0.01). Invasive ductal carcinoma was more common in men than in women (87.6% vs. 81.3%, p < 0.01), as was papillary BC (4.4% vs. 0.7%, p < 0.01). By contrast, the classic lobular subtype was more common in women (1.2% vs. 8.2%, p < 0.01) (3, 18). Most tumors were hormone receptor-positive and histological grade 2 (19). The 5-year overall survival (OS) was lower in men than in women (82.8% vs. 88.5%), and the risk of death from BC was 43.0% higher in men. This difference cannot be explained by diagnosis at advanced stages, because even among patients with stage I and II disease, men had worse OS than women (82.0% and 61.0% in men, respectively, and 90.0% and 79.0% in women, respectively) (3, 20, 21).

### Symptoms

The common complaints associated with FBC are often addressed in cancer prevention campaigns and are among the main motivations for seeking medical evaluation. However, as male breast complaints are rare, they are rarely commented upon, so they appear as a concern only in their occurrence, not as reasons for preventive conduct. As in women, breast complaints in men, including pain or enlargement of the mammary gland, are generally associated with benign issues (gynecomastia secondary to the use of medication) or physiological issues (teenager or senile gynecomastia). However, the presence of breast complaints, as well as misinformation on MaBC, can cause great discomfort and fear about the diagnosis (Table-1) (22, 23).

**Table 1:** Possible causes of gynecomastia in men (22, 23).

AGE-RELATED Newborns, puberty, elderly	FAT-RELATED Obesity, increased body fat
IDIOPATHIC	END-ORGAN ABNORMALITY
DRUG-RELATED	
Cannabis abuse; Use of anabolic steroids; occupational exposure to embalming fluids or oral contraceptives; contact with environmental phytoestrogens or phthalates;	
amlodipine, atorvastatin, benserazide, captopril, cimetidine, cladribine, combination, cytotoxic agents, cyclosporine, dasatinib, diazepam, didanosine, diethylproprion, digoxin, diltiazem, domperidone, D-penicillamine, etritinate, ffavirenz (HIV), fenofibrate, finasteride, fluoresone, fluoxetine, gabapentin, HAART, imatinib, indinaver (HIV), isoniazid, ketoconazole, marinol, methotrexate, metronidazole, nettle, nifedipine, omeprazole, paroxetine, phenytoin, pregabalin, ranitidine, rosuvastatin, saquinavir (HIV), spironolactone, stavudine, sulindac, sulperide, sunitinib, tandospirone, thalidomide, theophylline, venlafaxine, verapamil, vincristine.	

**HIV** = Human Immunodeficiency Virus; **HAART** = antiretroviral therapy

The main symptom of MaBC is the presence of a painless retroareolar mass (12). Depending on the time of disease onset and evolution, other symptoms may occur, such as nipple retraction, skin ulceration, local bleeding, and palpable axillary lymph nodes. However, imaging tests at the time of symptom onset are essential for the differential diagnosis or confirmation of the suspicion of MaBC.

The current recommendation of the American College of Radiology (ACR) for the assessment of palpable changes in the male breast differs in relation to the age at onset of symptoms (24). In patients younger than 25 years, ultrasonography should be the imaging method for the initial assessment. In patients older than 25 years, mammography should be the initial imaging method, followed by ultrasonography if the mammographic findings are inconclusive or suspicious of malignancy. Magnetic resonance imaging (MRI) is usually of little use in the routine investigation of changes in the male breast and should be restricted to individual cases, especially in the staging of confirmed malignant neoplasms.

The main differential diagnosis is gynecomastia, which can be differentiated from carcinoma using imaging methods. As fibroadenomas and cysts are very rare in men due to the lack of development of terminal ducto-lobular units in the male breast, any solid mass identified on imaging should be considered suspicious for malignancy (BI-RADS 4). In these cases, ultrasonography-guided percutaneous biopsy followed by histological examination of the biopsy specimen is necessary. Because of the small breast dimensions, the indication for mammography-guided biopsy is very rare (24).

MaBC usually presents on mammography as irregular, dense masses, with microlobulated, indistinct or spiculated margins, and eccentric in relation to the nipple-areola complex. Calcifications are less common than in women and present in approximately 30% of cases. On ultrasonography, the typical appearance is hypoechoic, solid, round or oval masses, with non-circumscribed margins (Figure-1). The higher frequency of papillary carcinomas results in a greater number of complex solid-cystic masses on ultrasonography (25).

### Therapeutic Approaches to Breast Cancer in Men

The treatment of BC is multidisciplinary, and recognition of the histopathological subtype, as well as the initial staging, are the first steps toward optimal treatment.

### Surgery

Surgical treatment of MaBC, whether upfront or after neoadjuvant chemotherapy, generally employs total mastectomy combined with sentinel lymph node dissection or axillary emptying because of the small breast volume and the presence of tumors close to the nipple-areola complex in most cases. Leone et al. (20) found that even patients with early-stage locoregional disease (81.2%) or tumors smaller than 1 cm (T1a or T1b) (74.0%) preferentially underwent total mastectomy. However, there was no difference in OS between patients undergoing mastectomy and those undergoing conservative surgeries (Table-2).

**Table 2:** Studies of MaBC surgical treatment.

	Mastectomy	Conservative Surgery
Srour et al., 2020, 49 cases (12)	87.8%	4.1%
Yadav et al., 2018, 81 cases (19)	86.0%	14.0%
Leone et al., 2016, 1263 cases (20)	81.2%	17.6%
Campos et al. 2021, 65 cases (unpublished data)	89.0%	4.6%

**MaBC** = male breast cancer

The indication for sentinel lymph node dissection in these patients follows the recommendation for FBC, i.e., patients with no initial clinical or radiological signs of axillary metastasis (N0) or those with N1 disease with clinical and radiological complete response after neoadjuvant chemotherapy. According to Leone et al., only patients who did not undergo axillary dissection had poorer survival (20).

An ongoing study with patients treated at the *A.C.Camargo Cancer Center* between 2000 and 2021 included 65 men with MC, 61 of whom underwent surgical treatment, including total mastectomy (58/61) and breast-conserving surgery (3/61). Of the patients for whom we had information about the axillary approach, 38 underwent axillary dissection and 21 underwent exclusive resection of the sentinel lymph node (unpublished data).

### Adjuvant therapy

Because of the rarity of MaBC, there are no robust clinical studies evaluating the role of systemic therapies in the management of both localized and metastatic disease. Hence, in current clinical practice, treatment strategies for men with BC are extrapolated from information arising from studies of FBC (26-28).

Since most BCs in men are hormone receptor-positive, endocrine therapy is the mainstay of treatment in these patients. The effectiveness of hormone deprivation, which initially involves orchiectomy, has long been established in the metastatic disease setting (5). Currently, the medication of choice to block estrogenic action is tamoxifen, a selective estrogen receptor modulator (26). In the adjuvant setting, observational studies show a benefit in OS with tamoxifen for at least 5 years after surgery (29). A recent meta-analysis with real-world data showed a significant increase in OS for patients receiving tamoxifen (HR 0.62, 95% confidence interval [CI] 0.41-0.95) (30). Therefore, for men with stage I–III hormone receptor-positive BC undergoing surgical treatment, adjuvant tamoxifen for 5 years is the therapy of choice, and it can be individually extended to 10 years based on tolerance and the risk of recurrence (26, 31).

Aromatase inhibitors (anastrozole, letrozole, and exemestane) perform better in the adjuvant setting than tamoxifen for postmenopausal women and are therefore the standard adjuvant therapies for FBC (29). However, their effectiveness is less evident in men. Population-based studies show poorer survival for men with BC treated with adjuvant aromatase inhibitors compared to those who received tamoxifen (29). Therefore, aromatase inhibitors should not be the routine choice in this setting, although they can be administered in combination with GnRH agonists or antagonists in patients with contraindications to tamoxifen (26).

Adjuvant endocrine therapy in men with BC has potential adverse effects that, as in women, can lead to treatment dropout. Up to 50% of men do not complete tamoxifen for 5 years after surgery. Sexual dysfunction/loss of libido and weight gain are the most common adverse effects, and hot flashes, mood alterations/depression, cognitive deficits, and thrombotic events may also occur (32).

Although the role of adjuvant chemotherapy in MaBC has not been established by prospective randomized clinical studies, observational studies have shown that it increases OS (33, 34). However, not all patients benefit from adjuvant chemotherapy. A study of 514 men with stage I–III BC in the Surveillance, Epidemiology, and End Results (SEER) database concluded that chemotherapy may not be necessary for patients with stage I and IIA disease (34). In general, the indications for adjuvant chemotherapy should take into account, especially, the tumor size, the histological grade, and the lymph node involvement. As in FBC, the use of the 21-gene recurrence score for male patients with hormone receptor-positive T1–T3/N0–N1 tumors may be useful to guide the decision regarding the administration of chemotherapy (26).

Treatments developed with the aim of overcoming endocrine resistance primarily in the female population, such as fulvestrant, mTOR inhibitors, and more recently CDK4/6 inhibitors and PI3K inhibitors, have also been indicated for the treatment of men with BC (26, 27, 35). In the rare cases of HER2-positive and triple-negative MaBCs, targeted therapies with anti-HER2 and anti-PD1 monoclonal antibodies, respectively, follow the same treatment recommendations for women with these tumor subtypes (26).

Considering the non-negligible prevalence of pathogenic germline variants in genes involved in DNA repair mechanisms in men with BC, especially *BRCA2*, the use of poly(adenosine diphosphate-ribose) polymerase inhibitors (PARPi) is of particular interest. In patients with these alterations, PARPi (olaparib and talazoparib) are an important therapeutic option for metastatic disease (36, 37). Recently, a phase 3 study of 1836 patients, including 6 men, with high clinical-risk BC and germline variant pathogenic or likely pathogenic mutations in *BRCA1/2* had increased invasive disease-free survival with adjuvant olaparib for 1 year (38).

## SPECIAL SITUATIONS FOR UROLOGISTS

### Testicular Tumors and Gynecomastia

Gynecomastia could be a first presentation in several neoplasms (39):

✓ Testicular: originating from germ (secreting forms), Leydig or Sertoli Cells;
✓ Adrenal: androgen - or estrogen - secreting tumors; mainly carcinomas (gynecomastia usually of recent onset and progress rapidly);
✓ Ectopic production of HCG (human chorionic gonadotropin), such as choriocarcinoma;

Leydig cell tumors are the most common of the 5% of sex cord-stromal tumors; they are generally benign lesions, with only 5-10% being considered malignant. Due to Leydig cells’ hormonally active properties, they can present with gynecomastia (the most common hormone-related presentation), precocious puberty, breast tenderness, reduce libido, erectile dysfunction, azoospermia, primary infertility, or even Cushing syndrome (40).

Sertoli cells tumors are often hormonally active, secreting estrogen. Males present with gynecomastia, advanced bone age, and rapid growth with short stature (41). These tumors typically emerge in syndromes such as Peutz-Jeghers (39).

In the case of enlargement of the breast and palpable glandular breast tissue, it is necessary to investigate the suspected tumor, through testicular palpation, ultrasound and referral to a urologist. Likewise, breast tissue hard, non-tender and/or joining underlying structures, the need for biopsy, referral to a breast cancer specialist and oncologic treatment (if malignant) should be evaluated (39).

### Prostate Cancer and Breast Cancer

Steroid hormones play an important role in the tumorigenesis of both MBC and prostate cancer. Prostate cancer is androgen-dependent and responsive to androgen blockade, whereas MaBC is estrogen-dependent and responsive to estrogen blockade in the vast majority of cases (19). Despite this divergence regarding hormonal risk factors, a possible relationship between prostate cancer and MaBC has been hypothesized for several decades (42).

Men with BC are at an increased risk of developing second primary neoplasms. In a multicenter study that included 3409 men with BC, 426 (12.5%) developed a second cancer (42). Small bowel cancer was the most common second cancer (standardized incidence ratio [SIR] = 4.95), followed by myeloid leukemia (SIR = 3.42). The SIR for prostate cancer was 1.61 (95% CI 1.34-1.93). Another study that analyzed data from 1788 men with BC from the SEER database found a non-significant increase in the incidence of prostate cancer (SIR = 1.09; 95% CI 0.85-1.37) (43), which is in agreement with the results of more recent studies [44]. Absolute data show that the incidence of prostate cancer in men with previously diagnosed BC ranges from 3.5% to 17.4% (Table-3), which is higher than that in the general population.

**Table 3:** Studies evaluating the incidence of prostate cancer in male patients with breast cancer.

Author, year	Institution/Data Source	Period	Number of participants with MBC	Patients with PC (%)	Patients with prior PC
Lee et al., 2009 (42)	Cleveland Clinic	1990–2006	69	12 (17.4)	6
Leibowitz et al., 2003 (45)	Dana-Farber Cancer Institute	1977–2000	161	10 (6.2)	2
Hemminki et al., 2005 (46)	Multi-institutional	1941–1997* 1978–1998	3409	119 (3.5)	0
Dawood et al., 2016 (47)	SEER	1990–2012	6970	644 (9.2)	NI
Satram-Hoang et al., 2006 (48)	California Cancer Registry	1988–2003	1926	69 (3.6)	0
Campos et al., ongoing**	Brazilian Cancer Center	2000–2021	65	11 (16.9)	3

**MaBC** = male breast cancer; **PC** = prostate cancer; **SEER** = Surveillance, Epidemiology, and End Results; **NI** = not informed.

* The period varied according to the institution. The longest and shortest periods are presented.

** Ongoing study, unpublished data.

BC accounts for approximately 0.09% of second primary cancers in men previously diagnosed with prostatic adenocarcinoma (48, 49). A 2003 study that analyzed data from all prostate cancer patients in the Swedish Cancer Registry (135,713 men from 1958 to 1996) showed an increased risk of MaBC (SIR = 2.01, 95% CI 1.44–2.74) (50). The authors concluded that this increased risk of BC after the diagnosis of prostate cancer could be explained by the estrogen therapy administered in the treatment of these patients, although this therapy is in decline. More recent population-based studies have not found an increased association of BC in men previously diagnosed with prostate cancer and instead show an overall lower incidence of a second neoplasm in prostate cancer patients (50, 51).

### Risk of BC in transgender patients

Little is known about the risk of BC in transgender patients because of the small number of studies, the underestimation of the number of cases, and the lack of recommendations for specific screening. However, BC diagnoses in transgender individuals are becoming more common, and knowledge on the subject is necessary for proper care (52). To properly discuss BC in these patients, it is important to define the following terms:

– **Cisgender man (or cis man):** an individual designated male at birth who identifies and lives as someone of the male gender.
– **Cisgender woman (or cis woman):** an individual designated female at birth who identifies and lives as someone of the female gender.
– **Transgender man (or trans man):** an individual designated female at birth who identifies and lives as someone of the male gender.
– **A transgender woman (or trans woman):** an individual designated male at birth who identifies and lives as someone of the female gender (53).

### BC in trans men

The risk of BC in these patients is considered to be similar to the risk of BC in cis men. Trans men frequently undergo breast reduction surgeries, similar to risk-reducing surgeries for patients with hereditary syndromes, and these surgeries, in addition to the administration of testosterone, reduce the risk of BC by approximately 90% (54). However, those who do not undergo breast reduction should be advised to maintain mammographic screening as indicated for women, with an annual mammogram starting at age 40. The recommendation is to follow the premise of “always track and track what you have” (53).

### BC in trans women

It is expected that the risk of BC in this group is higher than FBC, as these patients receive female hormones; however, this increase does not reach the risk of BC in cis women (54). There is an estimated 46-fold increase in risk of BC for trans women compared to cis men, but their risk is still lower than that of cis women, most likely explained by lower hormone levels (albeit prolonged hormone exposure) (55).

In relation to screening, despite the insufficient literature on the subject, biannual mammography is indicated from the age of 50 onwards, or from 5 to 10 years after the start of female hormone administration. We also lack data to support case management of trans women with a *BRCA1* mutation. However, a *BRCA1* mutation increases the risk of BC 6% in cis men and more than 78% in cis women. Therefore, if the patient does not want preventive removal of the breasts, they should be screened as high-risk cis women (56) (Table-4).

**Table 4:** BC risk and screening for trans men and women (13, 53-55).

Subject	Risk	Management
Cis men	1:1000; 1% of cancer cases in the male population	Not indicated
Cis wmen	1:8; 10-12% lifetime risk	Annual mammogram beginning at age 40
Cis men with BRCA mutation	> 6%	If the patient has gynecomastia, screening starts at age 50 or 10 years before the age of onset in the youngest individual in the family
Cis women with BRCA mutation	>78%	Earlier-onset mammography according to the type of mutation, and MRI is included in the screening
Trans men with both breasts	Lower risk than cis women due to the administration of testosterone	Biannual mammogram beginning at age 50; annual recommendation from the age of 40 [13].
Trans men without breasts (after mastectomy)	Risk similar to that of cis men because the breasts were removed	Mammography does not need to be performed; encourage education and self-knowledge
Trans women	46 times higher risk than cis men	Biannual mammogram beginning at age 50 or from 5-10 years of the start of female hormones

**BC** = breast cancer; **BRCA** = Breast Cancer Gene; **MRI** = Magnetic Resonance Imaging

## CONCLUSIONS

Despite its rarity, MaBC represents an important problem in men’s health that can be neglected if professionals who have higher access to this population are uninformed. Therefore, urologists can play an important role in the early diagnosis of MaBC because their work involves a broader scenario in which the focus is greater than sexual dysfunction and screening for prostate cancer. The promotion of care, whether for cis men, trans men, or their families, is the responsibility of these professionals.
